# Supplementary material for: O-GlcNAcylation of MITF regulates its activity and CDK4/6 inhibitor resistance in breast cancer
Source: Res Sq. 2023 Oct 3:rs.3.rs-3377962. Preprint. [Version 1] doi: 10.21203/rs.3.rs-3377962/v1 (PMC10602086; doi:10.21203/rs.3.rs-3377962/v1)
Supplement: Supplement 1 [file NIHPPrs3377962v1-supplement-1.pdf]

Supplementary Files

This is a list of supplementary files associated with this preprint. Click to download.

- SupplementaryTable1.xlsx
- SupplementaryTable2.xlsx
- SupplementaryTable3.xlsx
- Supplmentaldata.docx
